# Chronic fatigue syndrome in women assessed with combined cardiac magnetic resonance imaging

**DOI:** 10.1007/s12471-016-0885-8

**Published:** 2016-08-25

**Authors:** M. A. G. M. Olimulder, M. A. Galjee, L. J. Wagenaar, J. van Es, J. van der Palen, F. C. Visser, R. C. W. Vermeulen, C. von Birgelen

**Affiliations:** 1 Department of Cardiology, Thoraxcentrum Twente, Medisch Spectrum Twente, Enschede, The Netherlands; 2 Department of Epidemiology, Medisch Spectrum Twente, Enschede, The Netherlands; 3 Centre for Chronic Fatigue Syndrome, Amsterdam, The Netherlands; 4 Department of Research Methodology, Measurement & Data Analysis, University of Twente, Enschede, The Netherlands; 5 Department of Health Technology and Services Research, MIRA-Institute for Biomedical Technology & Technical Medicine, University of Twente, Enschede, The Netherlands

**Keywords:** Cardiovascular magnetic resonance, Chronic fatigue syndrome, Contrast enhancement, Left ventricular function, Myocardial damage

## Abstract

**Objective:**

In chronic fatigue syndrome (CFS), only a few imaging and histopathological studies have previously assessed *either* cardiac dimensions/function *or* myocardial tissue, suggesting smaller left ventricular (LV) dimensions, LV wall motion abnormalities and occasionally viral persistence that may lead to cardiomyopathy. The present study with cardiac magnetic resonance (CMR) imaging is the first to use a contrast-enhanced approach to assess cardiac involvement, including tissue characterisation of the LV wall.

**Methods:**

CMR measurements of 12 female CFS patients were compared with data of 36 age-matched, healthy female controls. With cine imaging, LV volumes, ejection fraction (EF), mass, and wall motion abnormalities were assessed. T2-weighted images were analysed for increased signal intensity, reflecting oedema (i. e. inflammation). In addition, the presence of contrast enhancement, reflecting fibrosis (i. e. myocardial damage), was analysed.

**Results:**

When comparing CFS patients and healthy controls, LVEF (57.9 ± 4.3 % vs. 63.7 ± 3.7 %; *p* < 0.01), end-diastolic diameter (44 ± 3.7 mm vs. 49 ± 3.7 mm; *p* < 0.01), as well as body surface area corrected LV end-diastolic volume (77.5 ± 6.2 ml/m^2^ vs. 86.0 ± 9.3 ml/m^2^; *p* < 0.01), stroke volume (44.9 ± 4.5 ml/m^2^ vs. 54.9 ± 6.3 ml/m^2^; *p* < 0.001), and mass (39.8 ± 6.5 g/m^2^ vs. 49.6 ± 7.1 g/m^2^; *p* = 0.02) were significantly lower in patients. Wall motion abnormalities were observed in four patients and contrast enhancement (fibrosis) in three; none of the controls showed wall motion abnormalities or contrast enhancement. None of the patients or controls showed increased signal intensity on the T2-weighted images.

**Conclusion:**

In patients with CFS, CMR demonstrated lower LV dimensions and a mildly reduced LV function. The presence of myocardial fibrosis in some CFS patients suggests that CMR assessment of cardiac involvement is warranted as part of the scientific exploration, which may imply serial non-invasive examinations.

## Introduction

Chronic fatigue syndrome (CFS) is relatively common with an estimated worldwide prevalence of 0.4–1 % [1]. The diagnosis of CFS relies on the presence of unexplained persistent or relapsing chronic fatigue in combination with a cluster of clinical symptoms [1–3]. Various potential aetiologies have been discussed. Ongoing research suggests that latent (chronic active) myocardial infections with Epstein-Barr virus or human cytomegalovirus may trigger CFS, and myocardial fibrosis has been described in this setting [4–6]. In CFS, cardiac involvement has been reported with reduced left ventricular (LV) dimensions, decreased left ventricular ejection fraction (LVEF), LV wall motion abnormalities, and cardiomyopathies with virus persistence in the myocardium [7–10].

Cardiac magnetic resonance (CMR) imaging may be useful for the non-invasive assessment of cardiac involvement in patients with CFS. This technique permits the examination of cardiac morphology and function, and even tissue characterisation of the LV wall to identify oedema or myocardial fibrosis – the consequences of myocardial inflammation [11]. Contrast-enhancement CMR imaging allows the identification of myocardial fibrosis. T2-weighted imaging, on the other hand, allows the visualisation of myocardial oedema and may be useful for staging myocarditis [12]. In the only other study with CMR imaging in the setting of CFS, 12 patients and 10 matched controls were assessed with cine and tagging imaging, but without the use of the contrast enhancement technique (i. e. without tissue characterisation) [7].

Therefore, in the present study with CMR imaging in patients with confirmed CFS, we used a combined approach of cine, contrast-enhanced, and T2-weighted imaging to assess LV tissue characteristics in addition to cardiac dimensions and function. Data from 12 patients with CFS were then compared with data from an age- and gender-matched group of 36 healthy controls, who were examined with the same imaging protocol.

## Methods

### Study population

We studied 48 females with a combined CMR approach. A consecutive series of 12 patients with CFS were recruited from a specialised CFS centre, to be examined with CMR. The diagnosis of CFS was based on the revised case definition by Fukuda et al. [3] after ruling out other potential causal diseases. Measurements in the 12 CFS patients were then compared with 36 age-matched, healthy female volunteers without any comorbidities (control group), who provided informed consent prior to CMR examination. The study complied with the Declaration of Helsinki for investigation in human beings, and was performed after approval and supervision of our institutional ethics committee.

### CMR data acquisition

CMR examination was performed on a 1.5-T whole body scanner (Achieva scan, Philips Medical Systems, Best, the Netherlands) using commercially available cardiac CMR software. For signal reception, a five-element cardiac synergy coil was used. Electrocardiogram triggering was done with a vector electrocardiogram setup. Subjects were examined in the supine position. Morphological images in the standard views were acquired by using fast field echo cine images (slice thickness 8.0 mm; repetition time 3.4 ms; echo time 1.7 ms; flip angle 60°; matrix 256 × 256).

Subsequently, a breath-hold, black-blood, T2-weighted double-inversion recovery sequence with a fat-saturation pulse was performed in 8–10 short-axis slices, with the following parameters: repetition time 1800–2400 ms; echo time 80 ms; matrix 256 × 256; field of view 32–40 cm; slice thickness 12 mm; number of excitations 1. Myocardial scar was assessed on contrast-enhanced multislice standard views, obtained approximately 10 minutes after intravenous bolus injection of 0.2 mmol gadolinium per kilogram body weight (Shering AG, Berlin, Germany). A three-dimensional Turbo Field Echo inversion recovery T1-weighted sequence was used with the following parameters: repetition time 4.0 ms; echo time 1.3 ms; flip angle 15°; inversion time individually optimised to null myocardial signal (usually between 180–250 ms); matrix 157; slice thickness 10–12 mm).

### CMR data analysis and definitions

CMR data were analysed on a workstation using dedicated software for cardiac analysis (Philips MR workspace, Release 2.5.3.0 2007-12-03; Philips, the Netherlands). Left ventricular end-diastolic (ml) and end-systolic volumes (ml), stroke volume (ml), LVEF (%) cardiac output (l/min), and end-diastolic wall mass (g) were calculated from contiguous short-axis loops by segmentation of endocardial and epicardial borders on each frame. Papillary muscles were regarded as part of the ventricular cavity.

The left ventricular wall regions were divided into 17 segments according to a standardised myocardial segmentation model [13]. Normal wall motion was assigned a score of 0, mild hypokinesia 1, severe hypokinesia 2, akinesia 3, and dyskinesia as 4. The wall motion score index was calculated by dividing the sum of scores in each segment by the total number of observed segments. T2-weighted images were considered if abnormal increased signal intensity was visually observed within the myocardium.

The contrast-enhanced images were independently evaluated by three experienced observers, who were blinded to patient/control characteristics and to the evaluations of each other. Contrast enhancement was defined as a zone of hyperenhancement on the late contrast-enhanced images (in contrast with the dark-grey signal of the normal myocardium). The studies were considered abnormal when at least two independent observers described the same abnormalities with agreement in both the presence of contrast enhancement and its location (same segment), reproducible in more than one contiguous slice and in more than one projection.

### Statistical analysis

Continuous variables had a normal distribution and were expressed as mean ± standard error. Categorical data were expressed as frequencies and percentages. To compare our CMR findings in CFS patients, we used age- and gender-matched controls. Student’s t‑test and the Mann-Whitney U test were used to compare continuous variables, and chi-square test and Fisher’s exact test were used to compare categorical variables. A *p* value < 0.05 was considered statistically significant.

## Results

### Patient characteristics

Twelve female CFS patients and 36 age- and gender-matched controls were analysed in this study. The patients and controls were relatively young (36 ± 13 vs. 29 ± 8 years). All but one CFS patient had a history of infectious mononucleosis disease with IgG antibodies to Epstein-Barr virus or human cytomegalovirus capsid antigen; all of these eleven patients had antibodies to Epstein-Barr virus nuclear antigen. Demographics and baseline characteristics did not differ between both groups (see also Table 1).

**Table 1:** Baseline demographics of CFS patients and age-matched control group of normal, female subjects

Variables	CFS patients	Control group	*p* value
Age (years)	36 ± 3	29 ± 8	0.11
Female patients *n* (%)	12 (100 %)	36 (100 %)	NA
Body height (cm)	168 ± 6.6	171 ± 6.4	0.28
Body weight (kg)	60.3 ± 9.6	64.7 ± 8.8	0.16
BMI	21.3 ± 2.2	22.2 ± 2.5	0.24
BSA	1.7 ± 0.2	1.7 ± 0.3	0.78
HR (beats/min)	80 ± 16	70 ± 13	0.09
SBP (mm Hg)	133 ± 1	124 ± 17	0.18
DBP (mm Hg)	77 ± 8	75 ± 12	0.63

Data are expressed as mean ± standard deviation or frequencies and percentages

*BMI* body mass index, *BSA* body surface area, *HR* heart rate, *SBP* systolic blood pressure, *DBP* diastolic blood pressure, *NA* not applicable

### CMR results

**Fig. 1:**
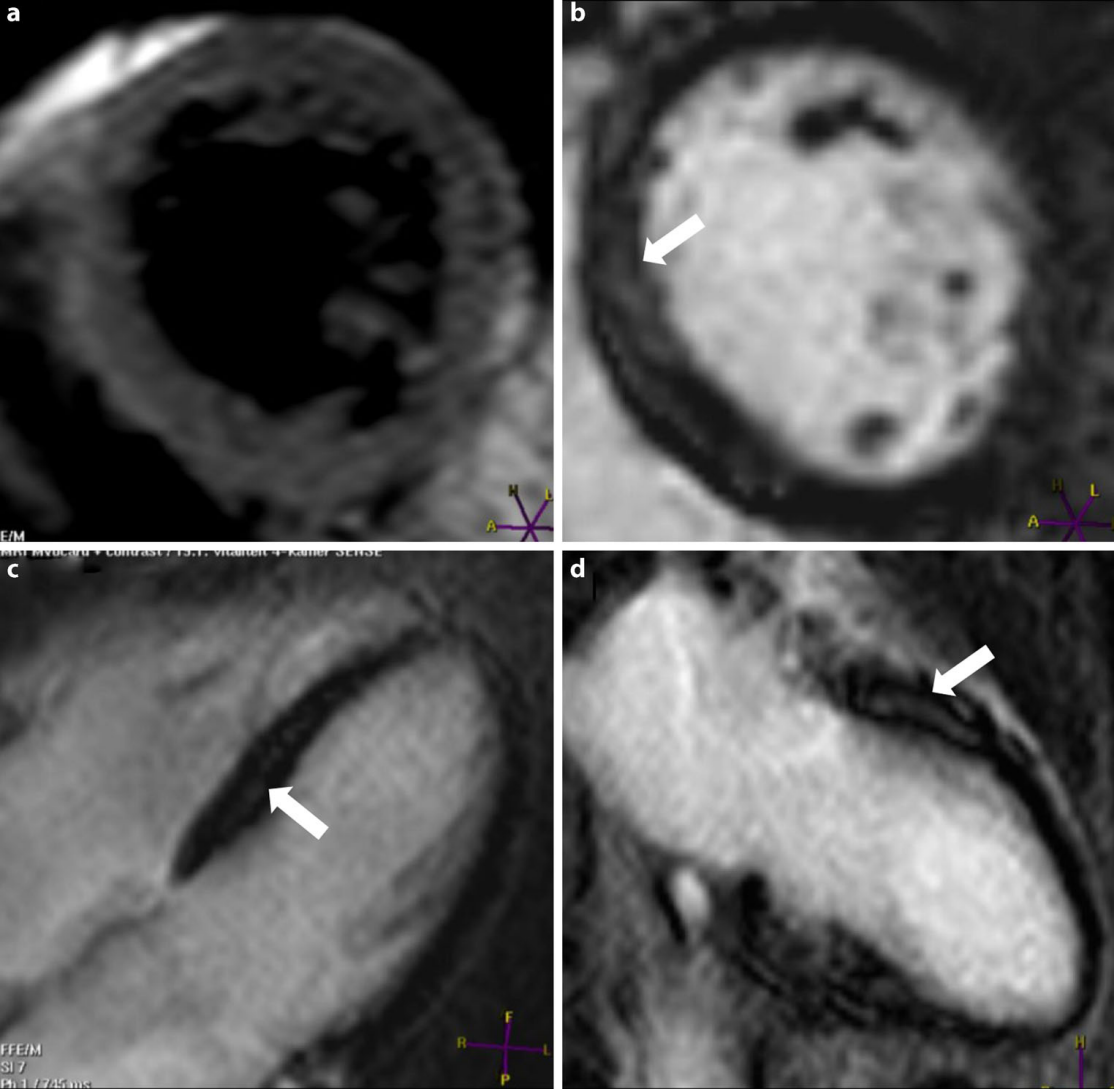
CMR findings in patient with chronic fatigue syndrome. Patient 1. **a** T2-weighted CMR imaging short-axis view; no presence of increased signal intensity was observed. **b,** **c,** **d** Contrast-enhanced-CMR imaging short-axis view, four-chamber view and two-chamber view; arrow demonstrates midwall contrast enhancement in the basal inferoseptal, septal and anteroseptal segments

The complete CMR examination protocol was followed in all CFS patients and controls; image sequences of a high quality could be obtained in all cases. CMR data of our CFS population are presented in Table 2. In CFS patients, LVEF (57.9 ± 4.3 %; *p* < 0.01), end-diastolic diameter (44 ± 3.7), as well as body surface area corrected end-diastolic LV volume (77.5 ± 6.2 ml/m^2^; *p* < 0.01), LV stroke volume (44.9 ± 4.5 ml/m^2^; *p* < 0.001), and LV wall mass (39.8 ± 6.5 g/m^2^; *p* = 0.02) were significantly lower than age- and gender-matched controls. Mild wall motion abnormalities were observed in four CFS patients in the basal and/or mid inferoseptal wall, leading to a wall motion score index of 0.02 ± 0.04; none of the controls showed wall motion abnormalities. Myocardial damage (i. e. fibrosis), as indicated by the presence of contrast enhancement, was observed in three patients who showed such lesions in the basal inferoseptal, septal, and anteroseptal midwall segments. An example is presented in Fig. 1. No contrast enhancement was seen in healthy controls. Only one patient showed both contrast enhancement and wall motion abnormalities. Regional or global oedema, as indicated by an increased signal intensity on T2-weighted images, was not observed in any of the patients.

**Table 2:** CMR findings in CFS patients and comparison with an age-matched control group of normal, female subjects

Variables	CFS patients	Control group	Mean difference + 95 % CI	*p* value
LVEDD (mm)	44 ± 3.7	49 ± 3.7	5 (2.68–7.66)	<0.05
EDV (ml)	129.8 ± 16.0	148.9 ± 19.0	19.1 (6.75–31.47)	<0.05
EDV/BSA (ml/m^2^)	77.5 ± 6.2	86.0 ± 9.3	8.4 (2.58–14.27)	<0.05
LVESD (mm)	29.7 ± 4.1	30.5 ± 3.2	0.8 (−1.48–3.18)	0.47
ESV (ml)	54.7 ± 10.1	54.0 ± 9.2	0.7 (−6.99–5.71)	0.84
ESV/BSA (ml/m^2^)	32.6 ± 4.0	30.9 ± 4.8	1.7 ± 1.6 (−4.92–1.48)	0.29
IVS (mm)	7.9 ± 0.9	8.1 ± 1.1	0.3 (−0.44–0.97)	0.46
EDWM (g)	60.6 ± 10.6	86.6 ± 11.7	26.0 (18.30–33.70)	<0.05
EDWM/BSA (g/m^2^)	39.8 ± 6.5	49.6 ± 7.1	9.9 (5.16–14.59)	<0.05
SV (ml)	75.0 ± 8.9	96.2 ± 11.7	21.2 (13.68–28.73)	<0.05
SV/BSA (ml/m^2^)	44.9 ± 4.5	54.9 ± 6.3	10.0 (6.07–14.03)	<0.05
CO (l/min)	4.9 ± 1.3	6.6 ± 1.2	1.7 (0.77–2.56)	<0.05
LVEF (%)	57.9 ± 4.3	63.7 ± 3.7	5.8 (3.19–8.41)	<0.05
WMSI	0.02 ± 0.04	0.00 ± 0.00	0.02 (−0.04–−0.01	0.054
T2 weighted oedema (presence, *n*)	0	NA	NA	–
CE (presence, *n*)	3	0	–	–

Data are expressed as mean ± standard deviation or frequencies and percentages

*CI* confidence interval, *LVEDD* left ventricular end-diastolic diameter, *EDV* end-diastolic volume, *BSA* body surface area, *LVESD* left ventricular end-systolic diameter, *ESV* end-systolic volume, *IVS* interventricular septum, *EDWM* end-diastolic wall mass, *SV* stroke volume, *SVI* stroke volume index, *CO* cardiac output, *LVEF* left ventricular ejection fraction, *WMSI* wall motion score index, *CE* contrast enhancement, *NA* not applicable

## Discussion

### Data of cardiac magnetic resonance imaging

The present CMR study used a combined CMR approach of cine, contrast enhancement, and T2-weighted imaging to assess cardiac dimensions, function, and myocardial tissue characteristics in patients with confirmed CFS and healthy controls. Our data demonstrate a left ventricular size, mass, and function that were lower than in age- and gender-matched controls, although still within the normal range. Theoretically, this difference could partially explain some mild fatigue in these patients, but it may also be suggested that the reduction in LV dimensions and function is the result of reduced physical activity. In this respect, Takenaka et al. found significantly reduced ventricular diastolic dimensions and cardiac output after 20 days of bed rest [14]. These findings are also in accordance with a previous echocardiographic study, which revealed a smaller LV chamber with a lower cardiac output in CFS patients during pharmacological stress testing [15].

Recently, Hollingsworth et al. investigated geometrical and functional LV parameters with CMR, including strain analysis, in 12 CFS patients and 10 healthy controls [7]. Although not all analyses gave evidence of myocardial dysfunction in CFS patients, residual torsion at 150 % of the end-systolic time was greater in CFS patients than controls, suggesting a delay in the release of torsion. In CFS patients, both residual torsion at 150 % of the end-systolic time and torsion to endocardial strain ratio correlated negatively with the end-diastolic volume index. These findings may contribute to the understanding of cardiac functional impairments in CFS patients [7].

In the present study, we also performed contrast-enhanced imaging in both CFS patients and a control group. We found no increase in signal intensity (i. e. oedema) on the T2-weighted CMR images, which demonstrates that our patients did not suffer from an active state of viral myocardial infection at the time of CMR. Midwall contrast enhancement, a finding that has frequently been observed in patients with histopathological evidence of chronic active or borderline myocarditis [16], was found in three out of 12 CFS patients. As demonstrated by Mahrholdt et al. in patients with acute myocarditis, contrast enhancement may not be detectable in approximately 25 % of patients at an average follow-up of 4.5 months [17]. Therefore, one may hypothesise that the initial myocardial involvement in our CFS patient population might have been somewhat larger than observed in our CMR examination during chronic state. The presence of a non-permissive, persistent viral infection, in which only a very low level of complete infectious virus is produced, could be a potential explanation of *isolated* midwall contrast enhancement (so, in the absence of oedema) [18] as observed in three of our CFS patients.

### Pattern and pathophysiology of myocardial damage

Previous myocarditis CMR studies demonstrated a relation between the type of virus and the pattern of myocardial damage [17]. Accordingly, the distribution of contrast enhancement may help to distinguish between different viral infections of the myocardium. In our CFS patients, contrast enhancement was exclusively observed in the infero- and antero-septum, which fits well with myocardial infections by Epstein-Barr virus [19], or human cytomegalovirus; serum antibodies for at least one of these viruses were identified in 11 of our CFS patients. Notably, inferoseptal wall motion abnormalities were observed in one patient with and three patients without contrast enhancement. In patients with myocarditis, the value and interpretation of contrast enhancement can be difficult because of the heterogeneous scar distribution and the generally lower signal intensities. In addition, due to limited voxel resolution, smaller myocardial scars may not be detected. Another explanation for this discrepancy could be the absence of contrast enhancement in the chronic state. To the best of our knowledge, this study is the first to suggest a septal predilection pattern of contrast enhancement and/or wall motion abnormalities in CFS patients. In CFS patients, myocardial fibrosis may lead to ventricular dysfunction, as in patients with Chagas myocarditis, for instance, in whom a relation between the amount of contrast enhancement and late ventricular dysfunction has been described [20].

### Clinical implications and consequences

In the present study, in patients with CFS, diagnosis was based on the diagnostic criteria of Fukuda et al. [3]. Future studies might also assess cardiac morphology and function in patients with myalgic encephalomyelitis and its diagnostic criteria [21]. Few data are available on prevalence and extent of cardiac involvement in CFS patients. With our combined CMR approach, we demonstrated in one examination (although in the normal range) lower size, mass, and function of the left ventricle and structural abnormalities of the myocardium, as previously assessed separately with different techniques. Specific treatment of myocarditis remains a challenge [6, 22]. A small, randomised, placebo-controlled trial on the use of antiviral therapy for patients with a Epstein-Barr virus subset of CFS found a clinical improvement after six months [23]. Recently, it has been suggested that Epstein-Barr virus can provoke ventricular tachycardia by both acute and chronic myocardial inflammation, emphasising the clinical importance of this viral infection [19]. To test the hypothesis that Epstein-Barr and/or human cytomegalovirus may lead to ventricular dysfunction, myocardial involvement, and CFS, further studies are warranted that should include early and repeat examinations with a combined CMR approach plus targeted myocardial tissue biopsies. Such serial studies may answer the question of whether some CFS patients develop a dilated cardiomyopathy with the inherent risk of an inferior clinical course [6].

### Limitations and technical considerations

We did not perform serial serological tests or myocardial biopsies. It would have been ideal to correlate CMR findings with targeted endomyocardial biopsies. However, this would have involved an invasive procedure without a direct therapeutic implication. In patients with myocarditis, the value and interpretation of contrast enhancement can be difficult because of heterogeneous scar distribution and the generally lower signal intensities [4, 24–26]. Although our data are unique, findings in this small population of CFS patients cannot answer the question whether the somewhat lower cardiac function may contribute to the reduced exercise capacity or may be a result of lower physical activity. Further assessment of relationships between exercise capacity and CMR parameters in a larger patient population is of interest and should be addressed by future studies.

## Conclusion

In patients with chronic fatigue syndrome, CMR demonstrated relatively lower dimensions and a mildly reduced function of the left ventricle. The presence of myocardial fibrosis in some CFS patients suggests that further assessment of cardiac involvement may be warranted as part of a further scientific exploration of the CFS disease. This may imply serial non-invasive examinations with CMR.
